# Surgical management of complex mediastinitis: an 8-year single-centre experience reinforcing the role of open thoracotomy

**DOI:** 10.1093/icvts/ivaf154

**Published:** 2025-06-27

**Authors:** Nabih Berjaoui, Felix Santos, Fabiana Curti, Puiyee Sophia Chan, Savvas Lampridis, Akshay Patel, Andrea Bille

**Affiliations:** Department of Thoracic Surgery, Guy’s Hospital, Guy’s and St Thomas’ NHS Foundation Trust, London, UK; King’s College Medical School, Great Maze Pond, London, SE1 1UL, UK; Department of Thoracic Surgery, Guy’s Hospital, Guy’s and St Thomas’ NHS Foundation Trust, London, UK; Department of Thoracic Surgery, Guy’s Hospital, Guy’s and St Thomas’ NHS Foundation Trust, London, UK; Department of Thoracic Surgery, 424 General Military Hospital, Thessaloniki, Greece; Department of Thoracic Surgery, Guy’s Hospital, Guy’s and St Thomas’ NHS Foundation Trust, London, UK; Institute of Immunology and Immunotherapy, University of Birmingham, Birmingham, B15 2TT, England; Department of Thoracic Surgery, Guy’s Hospital, Guy’s and St Thomas’ NHS Foundation Trust, London, UK

**Keywords:** mediastinitis, surgery, thoracotomy, video-assisted thoracoscopic surgery, VATS

## Abstract

**OBJECTIVES:**

Mediastinitis is an infection affecting the mediastinum, often caused by cardiovascular or thoracic surgery procedures. Management entails antibiotic therapy, surgical debridement, drainage of infected sites and immediate or delayed closure. Negative pressure wound therapy is useful in cases of delayed sternal closure. Several approaches for mediastinal drainage have been proposed, but there is no consensus on the thoracic intervention approach.

**METHODS:**

A single-centre, retrospective analysis from the UK analysed data from 19 patients who underwent surgical management for mediastinitis between September 2015 and April 2023. Our primary aim was to describe the outcomes from our series where we predominantly employed an open surgical approach.

**RESULTS:**

The mean age of our cohort was 49 ± 17.12 years old; the mean performance status (PS ECOG) was 2 ± 0.77. Two people were known smokers (10.53%), while five were non-smokers (26.31%). Fifteen patients underwent an open operation (78.85%), with rest undergoing a minimally invasive approach. The majority of procedures were undertaken from the right-hand side. The overall intensive care unit admission rate was 68.42% (*n* = 13) with an in-hospital complication rate of 5.26% (*n* = 1). This was a respiratory arrest secondary to mucous plugging. There were no in-hospital deaths, and median follow-up was 41 months (22–50). Overall survival at 3 years was 85%.

**CONCLUSIONS:**

Open thoracotomy remains an important surgical strategy in the management of complex mediastinitis, but further validation is required through larger, prospective studies.

## GRAPHICAL ABSTRACT

**Figure:**
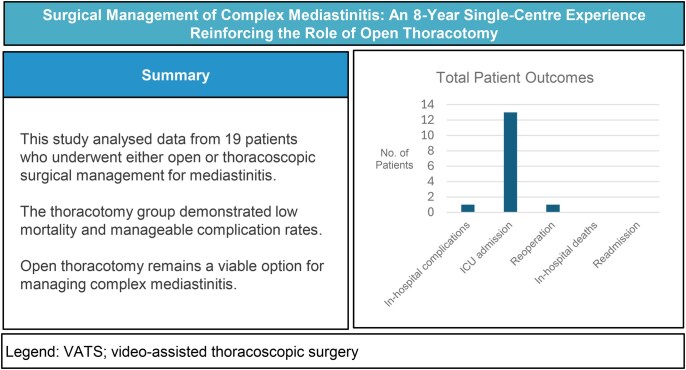

## INTRODUCTION

Mediastinitis is a rare but life-threatening condition that may result from deep sternal wound infection (DSWI), oesophageal perforation or descending necrotizing mediastinitis (DNM) [1]. DNM in particular carries a high mortality rate and arises from cervicofacial infections that descend into the mediastinum via fascial planes [2–5]. Prompt diagnosis and surgical drainage are critical to improving outcomes [5–7].

While minimally invasive strategies such as video-assisted thoracoscopic surgery (VATS) and robotic-assisted approaches are gaining popularity [8, 9], the role of open thoracotomy remains a subject of ongoing discussion. In certain cases of extensive or anatomically complex mediastinal infection, open access may allow for superior debridement, exposure and drainage.

This study presents an 8-year, single-centre experience focusing on the use of open thoracotomy in managing complex mediastinitis. We aim to describe its safety, efficacy and postoperative outcomes while providing a contextual overview of cases managed with non-open approaches.

## MATERIALS AND METHODS

### Ethical statement

This work did not require formal ethical approval as there was no direct patient contact. The collection and storage of data from research participants for multiple and indefinite use was consistent with the requirements outlined in the WMA Declaration of Taipei. The study was approved by the local research ethics committee who monitored ongoing use of databases.

### Cohort

This research was conducted as a single-centre, retrospective cohort study. Between September 2015 and April 2023, we collected the data of 19 consecutive patients who underwent surgery for the treatment of mediastinitis at Guy’s Hospital in London, a tertiary centre that specializes in thoracic surgery. All cases met the criteria by proposed by Estrera *et al.* in 1983 [10] for the diagnosis of this disease. These criteria included: the clinical manifestations of severe infection, demonstration of characteristic radiographic findings, documentation of necrotizing mediastinal infection at operation and establishment of the relationship of oropharyngeal or cervical infection with the development of the necrotizing mediastinal process.

Complex mediastinitis was defined as infection requiring thoracic operative intervention beyond cervical drainage, including Endo type IIa/IIb DNM and DSWI involving the mediastinum (type IV). Inclusion was based on radiological evidence of mediastinal extension, clinical sepsis and/or failure of conservative or less invasive surgical management. This ensured the cohort reflected anatomically extensive and clinically severe cases, consistent with established classification systems [1, 5, 11, 12].

**Figure 1:**
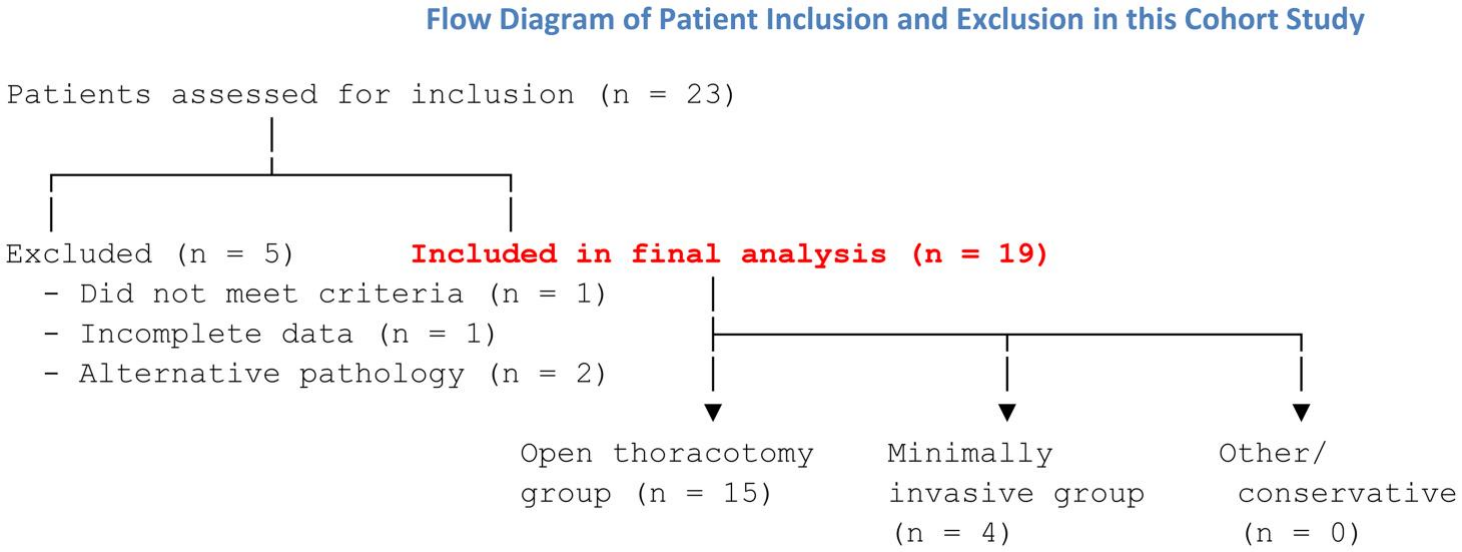
Flow diagram to illustrate patient inclusion in this cohort study

Excluded from the study were those patients who had mediastinitis secondary to other causes, such as infections after mid-sternotomy or after oesophageal rupture. Oesophageal perforation cases were excluded as their clinical course and surgical management are distinct from DNM. We analysed demographic variables, disease period, origin of infection, disease stage according to the endo classification, surgical technique performed and evolution, assessing survival and the appearance of complications. Figure 1 illustrates the cohort recruitment for this study.

### Operative techniques

Patients included in this series underwent surgical procedures employing the open and non-open techniques. Non-open included minimally invasive approaches, video mediastinoscopy or thoracoscopy or a combination of cervical mediastinoscopy and VATS. Thoracotomy, in which the surgeon makes an incision between two ribs to access the thorax, was the primary open approach.

Open thoracotomy was performed using a standard posterolateral thoracotomy approach in the majority of patients, with side selection based on the dominant anatomical extent of disease as identified on CT imaging. Usually, the fifth intercostal space was used to enter the chest. Thorough mediastinal debridement and washout were performed, with careful dissection through obliterated fascial planes to access infected compartments.

Large-bore chest drains were placed in both anterior and posterior spaces to allow continuous drainage. In cases with extensive soft tissue infection or where the wound was left open for further debridement, negative pressure wound therapy (NPWT) was employed using vacuum-assisted dressings to promote granulation and reduce infection load. Drain output, resolution of systemic inflammatory markers and clinical improvement guided drain removal and wound closure strategy.

### Data collection

Information was gathered about the age and sex of patients, ECOG performance status, smoking status, asbestos exposure, aetiology and type of mediastinitis, associated weight loss, comorbidities (pulmonary, cardiac, renal, endocrine and others), previous cancer, preoperative diagnosis, CT scan, date of surgery, approach, description of the procedure, amount of pleural effusion in ml and type of fluid, length of the operation in min, blood loss and units transfused, rib fractures at surgery, possible reoperation, microbiology, pathology, local anaesthesia, number of chest drains and length of stay of the drain, discharge date and length of postoperative stay in the hospital, in-hospital complications and their treatment, possible intensive care unit (ICU) admission and ICU stay, in-hospital deaths and readmissions.

This sample group is limited by its size, and we avoided direct statistical comparisons with the open group to prevent misinterpretation due to lack of statistical power; this includes Kaplan–Meier survival analysis that was further limited by the reliability of the long-term follow-up data. Instead, we have presented the outcomes for the minimally invasive group using descriptive analyses to provide insights without inferring any form of statistical interpretation which would be unreliable.

Our primary objective was to evaluate the safety and efficacy of the open approach in the management of complex mediastinitis. Our secondary objective was to analyse short- and long-term postoperative outcomes in our combined cohort overall and the long-term effectiveness of the open approach in preventing recurrence or progression. We additionally provide a descriptive analysis of minimally invasive approach cases. This study was not designed to provide a head-to-head comparison between the two approaches.

## RESULTS

The main characteristics of patients are summarized in Table 1. The mean age was 49 ± 17.12 years old; the mean performance status (PS ECOG) was 2 ± 0.77. Among the sample, two people were known smokers (10.53%), while five were non-smokers (26.31%); there were no data about the remaining subjects. Just one patient experienced weight loss (the one with thymoma). None of the subject had known pulmonary, cardiac or renal comorbidities, while two had diabetes (10.53%). One person had a previous primary lung cancer (adenocarcinoma T1bN0M0). Again, the comparison provided by this sample group was limited by its small size.

**Table 1:** Patient demographic, disease classification and operative characteristics

Patient characteristics	Type	Number of patients (*n*)	% of patients
Age	21–81	19	100.00
Gender	Male	13	72.22
	Female	5	27.77
Type of mediastinitis	Mediastinitis	7	36.84
	Descending mediastinitis	3	15.79
	Septic mediastinitis	2	10.53
	Mediastinitis post oesophageal perforation	1	5.26
	Retropharyngeal abscess	5	26.32
	Thymoma surgery	1	5.26
Approach	Thoracotomy (open)	15	78.85
	Mediastinoscopy	1	5.26
	Cervical incision	2	10.53
	Cervical incision and VATS	1	5.26
Laterality	Right	13	68.42
	Left	2	10.53
	Medial	3	15.79
	Bilateral	1	5.26

The mean amount of pleural effusion was 580.56 ± 566.24 ml. The type of effusion was purulent in 11 cases (57.89%), serous in 1 case (5.26%) and serous sanguinous in 1 case (5.26%); there were no data about the remaining cases. The average operating time was 101 min ± 54.25; the average blood loss was 180 ± 154.52 ml; the average number of units of blood transfused was 0.32 ± 0.67. One patient required reoperation with the purpose of cervical exploration to further control the mediastinal sepsis. There was one major in-hospital complication which was a respiratory arrest due to mucous plugging. This was managed with intubation, ventilatory support and flexible bronchoscopy. With regards to less severe complications, there were three incidences of wound infection not requiring debridement or vacuum-assisted closure (VAC), but anti-microbial therapy and lavage. Postoperative short-term outcome data are shown in Table 2.

**Table 2:** Total outcomes of surgeries and comparison between open and non-open approaches (mediastinoscopy, cervical incision, cervical incision and VATS)

	Total surgery outcomes		Open (*n* = 15)		Non-open (*n* = 4)	
Outcomes	Mean	Standard deviation	Mean	Standard deviation	Mean	Standard deviation
Number of chest drains	3.47	1.62	4.15	1.14	1.25	0.5
Length of chest drainage (days)	12.71	6.28	13.2	6.60	11.5	6.14
Length of stay (days)	30.57	23.82	33	24.71	14.5	7.78
OR time			116.25	49.23	40	17.32
Blood loss			216.67	153.14	42.5	43.49
Units of blood			0.4	0.74	0	0

	Number of patients (*n*)	% of patients	Number of patients (*n*)	% of patients	Number of patients (*n*)	% of patients
In-hospital complications	1	5.26	1	6.67	0	0
ICU admission rate	13	68.42	10	66.67	3	75
Reoperation	1		1	6.67	0	0
In-hospital deaths	0	0	0	0	0	0
Readmission	0	0	0	0	0	0

**Figure 2:**
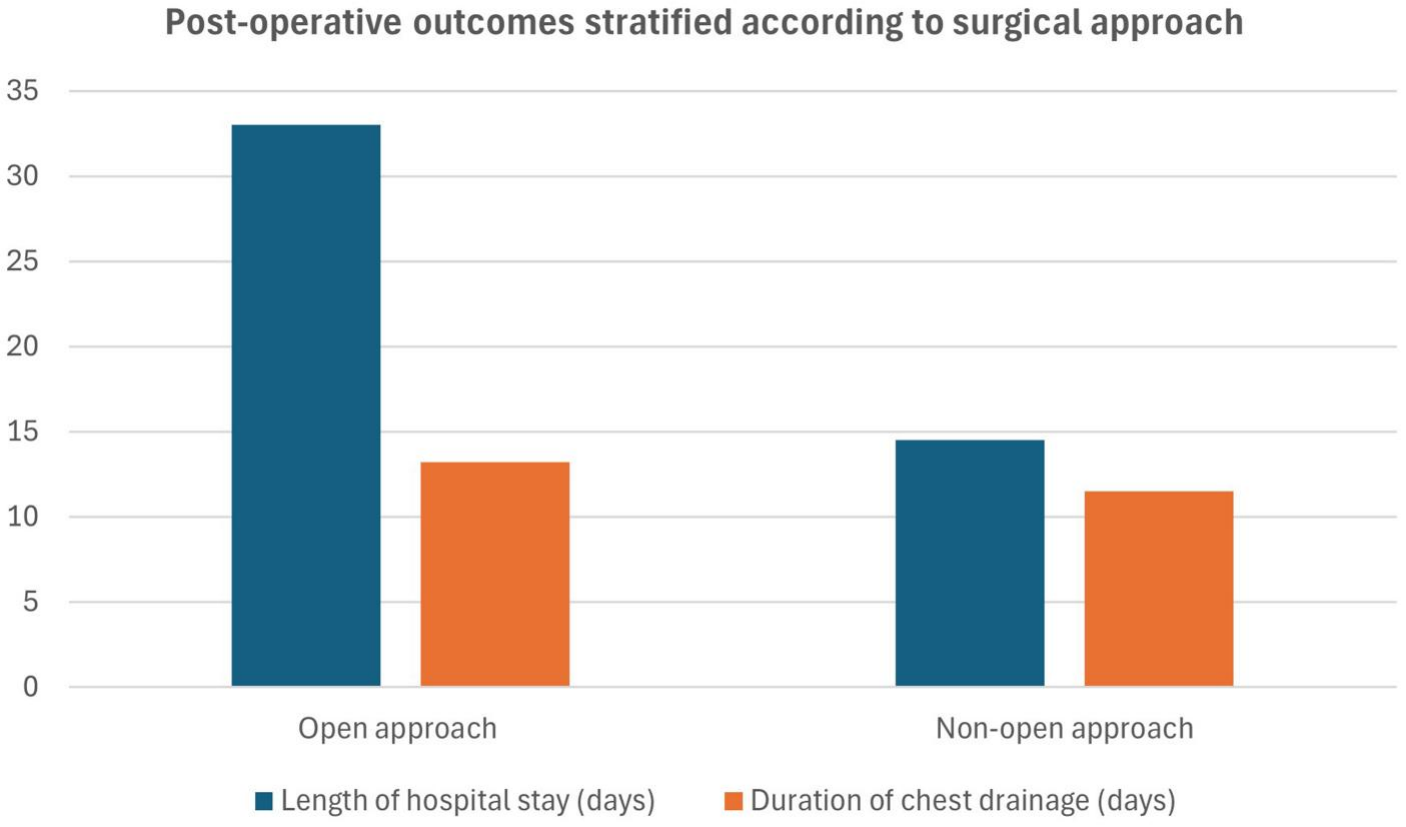
Bar chart illustrating length of hospital stay and length of chest drainage stratified according to surgical approach

We also examined the outcomes of the two subjects that had diabetes, since it is known entity associated with poor outcomes in this setting. In this case, the average pleural effusion was 500 ml ± 0; the average Operating Theatre time was 75 min ± 7.07; the average blood loss was 50 ml ± 70.71; the mean number of units of blood transfused was 1 ± 1.41. The average number of chest drains was 4 ± 1.41; the average length of chest drain duration was 6.5 days ± 2.12; the mean length of stay was 15.5 days ± 2.12. Figure 2 illustrates the length of hospital stay and length of chest drainage stratified according to surgical approach. They had no hospital complications (0%), and they were both admitted in ICU (100%). The median follow-up duration was 41 months (22–50). Overall survival at 3 years was 85% with deaths having occurred from cardiovascular causes and malignancy. There were no documented late recurrences of mediastinitis in our cohort.

### Microbiological analyses

Microbiological cultures were obtained from all patients at the time of surgery. The most frequently isolated organisms included *Staphylococcus aureus* (including Methicillin-Resistant Staphylococcus Aureus) (*n* = 6), *Streptococcus anginosus* group (*n* = 4), *Escherichia coli* (*n* = 3) and *Pseudomonas aeruginosa* (*n* = 2). Mixed anaerobes were grown in five patients and 80% of patients had polymicrobial infection involving oral flora, including anaerobes, reflecting odontogenic or pharyngeal sources. Antibiotic regimens were adjusted postoperatively based on culture sensitivities, with involvement of microbiology and infectious disease teams.

## DISCUSSION

Mediastinitis remains a life-threatening complication of thoracic and upper gastrointestinal pathology, with aetiologies that include DSWI, oesophageal perforation, anastomotic leak and DNM [1]. Our single-centre experience over 8 years demonstrates that open thoracotomy remains a safe and effective surgical approach in the management of complex mediastinitis. Among the 15 patients who underwent open thoracotomy, there were no in-hospital deaths or readmissions. Although comparative statistical analysis between the open and minimally invasive groups was limited by the small cohort sizes (15 vs 4), the clinical outcomes observed in the open group provide reassurance regarding the efficacy of this approach in appropriately selected patients.

The DSWI subset of mediastinitis, classified by depth and extent [11], is associated with a 0.5–2.2% incidence after cardiac surgery and a mortality rate of approximately 14% [1, 13]. Risk factors include diabetes, peripheral vascular disease, obesity, chronic obstructive pulmonary disease (COPD) and prolonged surgery [1, 4, 14]. Similarly, mediastinitis secondary to oesophageal perforation, whether spontaneous (e.g. Boerhaave’s syndrome), iatrogenic or anastomotic in origin, represents a high-risk cohort with significant morbidity [15–17]. The 30% anastomotic failure rate following oesophagectomy and the critical need for timely intervention highlight the challenges in this group [17–19].

DNM, an aggressive form of mediastinitis, occurs through fascial plane spread of oropharyngeal or odontogenic infection into the mediastinum [2–5]. The Endo classification delineates type I (upper mediastinum) from types IIa and IIb (anterior and posterior lower mediastinum, respectively), with thoracic intervention recommended for types IIa and IIb [12]. Delays in diagnosis, inadequate debridement and insufficient drainage are recognized contributors to the high mortality rate associated with DNM, estimated at up to 30% [5–7]. Risk factors include immunosuppression, diabetes, respiratory compromise and poor tissue perfusion [1, 5–7, 20]. In this context, the choice of surgical approach is important. See Table 3 for a concise summary of pertinent risk factors associated with the aetiopathogenesis of DNM and DSWI.

**Table 3:** Risk factors associated with DSWI and DNM

DSWI	DNM
Diabetes mellitus	Diabetes mellitus
Peripheral vascular disease	Immunosuppression
Obesity	Respiratory compromise
Chronic obstructive pulmonary disease (COPD)	Poor tissue perfusion
Prolonged surgery	Delayed diagnosis
	Inadequate debridement or drainage
	Severe oropharyngeal or odontogenic infection

The general principles of mediastinitis management include aggressive surgical debridement, drainage, empiric then culture-directed anti-microbial therapy and supportive care such as nutritional optimization and wound VAC [1, 21–23]. Open thoracotomy allows for comprehensive access to the mediastinum and pleural cavities, facilitating meticulous debridement across fascial planes and reducing the likelihood of residual sepsis or progression. Our data support this paradigm, especially in patients with extensive disease or unclear anatomical planes, where minimally invasive access may be insufficient.

The current era of thoracic surgery increasingly favours minimally invasive approaches (VATS, robotic) [8, 9], yet our study highlights the continued value of open thoracotomy in managing complex mediastinitis. The preference for thoracotomy in our cohort was based on several interrelated factors: anatomical extent of disease, degree of tissue necrosis, haemodynamic stability, patient co-morbidities (diabetes, smoking history and immunosuppression) and urgency of intervention.

In many cases, the infection had spread diffusely across multiple mediastinal compartments (Endo type IIa or IIb), with obliteration of tissue planes and evidence of empyema or multi-cavity contamination on imaging. These features often precluded safe or effective debridement via a minimally invasive route, where visibility and reach are inherently more limited. Additionally, several patients were haemodynamically unstable or had respiratory compromise, which necessitated prompt and definitive source control through a single-stage, wide-access approach rather than a staged or limited VATS procedure. In some cases, preoperative imaging underestimated the true extent of the mediastinal involvement, which was only fully appreciated intraoperatively justifying an open approach in retrospect.

The minimally invasive techniques are useful in early or localized disease, where there is a higher chance of optimal visualization, reliable working space and controlled dissection across inflamed or necrotic tissue beds, all of which may be compromised in advanced mediastinitis. The elevated reoperation rates observed in VATS series [24, 25] may reflect both case selection bias, where patients perceived to have ‘less severe’ disease are triaged for VATS but subsequently found to have inadequate drainage and technical limitations in clearing deep or multi-compartmental infection. Moreover, institutional variation in surgeon experience with minimally invasive mediastinal surgery may influence outcomes. For instance, complex posterior mediastinal debridement or infected oesophageal leak management through VATS can be highly technically demanding, and some centres may not have the subspecialist expertise to pursue this safely.

That said, we acknowledge that minimally invasive approaches have a valuable role in carefully selected patients, particularly in early Endo type I or IIa disease, or in stable patients with focal collections amenable to targeted drainage. Future practice may increasingly favour hybrid strategies that combine the diagnostic precision of VATS with the definitive clearance possible through limited open incisions. However, based on the characteristics of our cohort and institutional protocols, open thoracotomy was deemed the most appropriate and safe route to achieve source control in the majority of cases.

Ultimately, the choice of surgical approach must be individualized, taking into account not only the anatomical and infectious burden but also patient physiology, resource availability and surgeon experience. A multidisciplinary, protocolized approach remains essential to optimize outcomes in this high-risk population.

Our findings are consistent with published literature. Marty-Ané *et al.* reported favourable outcomes in a 10-year series of 12 DNM patients, with nine undergoing thoracotomy and a resulting mortality rate of 16.5% [26]. Chen *et al.* demonstrated that of 18 patients treated with VATS or transcervical drainage, four required reoperation and two suffered fatal outcomes [24]. Similarly, Tanaka *et al.*, in a retrospective analysis of 141 DNM patients, showed that while both VATS and thoracotomy were viable, VATS was associated with significantly higher rates of postoperative complications (53% vs 24.1%) and reoperation (37.9% vs 15.5%) [25]. These findings align with our observation that open thoracotomy allows for more definitive control of the septic focus in complex presentations.

Our study also reinforces that surgical decision-making should consider not only the anatomical classification and extent of disease but also patient-specific and procedural risk factors. These include immunocompromised status, time from symptom onset to intervention and extent of tissue necrosis [1, 4, 7, 14, 20]. In institutions where early diagnosis and rapid access to multidisciplinary care are available, an aggressive open approach may reduce the need for multiple staged procedures or rescue interventions following minimally invasive failure.

A key strength of this study is the detailed capture of outcomes in a rare but high-acuity clinical scenario. The long-term follow-up and granular analysis of surgical outcomes provide a valuable snapshot of contemporary practice, albeit within the confines of a retrospective, single-centre framework. This study is not designed to refute the evolving role of VATS or robotic-assisted thoracic surgery (RATS) in select cases [9] but rather to reaffirm the continued relevance of open thoracotomy for patients with advanced or anatomically complex disease.

### Limitations

We acknowledge several limitations inherent to this study. First, the small sample size, particularly over an extended 8-year period, significantly limits the statistical power of our findings and precludes meaningful comparative analysis between surgical approaches. As a result, any observed differences in outcomes, such as complication or reoperation rates, must be interpreted with caution and should not be viewed as establishing causality. The absence of a matched control group further restricts our ability to control for confounding variables, and we have avoided drawing definitive conclusions regarding the superiority of any one approach.

Additionally, this is a retrospective, single-centre case series, which may reflect local expertise, referral patterns and institutional practices that differ from other centres. These factors inherently limit the external validity and generalizability of our results. Finally, although our data provide a valuable snapshot of real-world clinical decision-making and outcomes, the rarity and heterogeneity of complex mediastinitis underscore the need for larger, prospective, multicentre studies to confirm and build upon our observations.

## CONCLUSION

In this single-centre study of 19 patients with complex mediastinitis, open thoracotomy proved to be an effective management option, demonstrating low mortality and manageable complication rates. Although patients often experienced extended hospital stays and ICU admissions, this approach enabled thorough infection control in a diverse patient population with complex presentations and remains an important surgical strategy in the management of complex mediastinitis, but further validation is required through larger, prospective studies.

## Data Availability

The data underlying this article cannot be shared publicly due to the privacy of the individual that participated in the study. The data will be shared on reasonable request to the corresponding author.

## ABBREVIATIONS

DNM: Descending necrotizing mediastinitis
DSWI: Deep sternal wound infection
ICU: Intensive care unit
VATS: Video-assisted thoracoscopic surgery
